# The Influence of Phosphogypsum Addition on Phosphorus Release in Biochemical Treatment of Sewage Sludge

**DOI:** 10.3390/ijerph15061269

**Published:** 2018-06-15

**Authors:** Yelizaveta Chernysh, Magdalena Balintova, Leonid Plyatsuk, Marian Holub, Stefan Demcak

**Affiliations:** 1 Department of Applied Ecology, Faculty of Technical Systems and Energy Efficient Technologies, Sumy State University, 2, Rymskogo-Korsakova st., 40007 Sumy, Ukraine; e.chernish@ssu.edu.ua (Y.C.); l.plyacuk@ecolog.sumdu.edu.ua (L.P.); 2 Institute of Environmental Engineering, Faculty of Civil Engineering, Technical University of Kosice, Vysokoskolska 4, 04200 Kosice, Slovakia; stefan.demcak@tuke.sk; 3 Laboratory of Excellent Research, Faculty of Civil Engineering, Technical University of Kosice, Park Komenskeho 10/A, 04200 Kosice, Slovakia; marian.holub@tuke.sk

**Keywords:** phosphorus compound, phosphorus-recovery technology, sewage sludge, phosphogypsum, COD

## Abstract

The paper is focused on the research of biochemical treatment of sewage sludge and phosphogypsum under sulphate-reducing conditions with a phosphorus release process. The theoretical foundations of the work were based on the biochemical formalization using the principles of autocatalysis of natural systems. During the experimental research for the control of physicochemical parameters of the process spectroquantic, X-ray fluorescence analysis and other techniques were used. A schematic model of the dephosphatation process under anaerobic stabilization of sewage sludge and phosphogypsum was developed. The increase of phosphogypsum dosage had a close correlation with the release of phosphate ions. At the stimulating action of the phosphogypsum additive, a 2.5–5.0-fold increase in soluble phosphate concentration was observed. The rational dose of phosphogypsum was determined. Along with an increase the ratio of COD (Chemical Oxygen Demand)/phosphogypsum to 0.1, an increase in the phosphate ions in solution was observed. A further increase in the ratio of COD/phosphogypsum did not affect the concentration of phosphate ions in solution.

## 1. Introduction

The complete phosphorus cycle in current human society is endangered because phosphorus is a non-renewable resource. Therefore, with depletion of its reserves, it is necessary to develop sustainable techniques for its recover from solid and liquid waste.

Due to the limited availability of phosphate rock, phosphorus recovery has attracted increasing interest from researchers. A proven technique to efficiently remove and recover phosphorus from phosphorus-rich streams is struvite precipitation [1,2,3]. However, the suitability of struvite precipitation for agricultural use is largely determined by the content of heavy metals and micropollutants [2].

The most widely used methods of P removal from wastewater are through biological means. The enhanced biological phosphorus removal (EBPR) method can be regarded as a two-stage process, i.e., an anaerobic and an aerobic phase [1,4].

EBPR integration in short sludge residence time (SRT) systems is not a straightforward issue since the need for an anaerobic phase hinders the possibility of working at very low SRTs. EBPR in full-scale wastewater treatment plants is usually operated at high SRTs (around 10 days) which is adequate to promote PAO growth [5]. Noted, the extracellular polymeric substances (EPS) variations under the anaerobic-aerobic process are closely related to the uptake and release of P in the biological phosphorus removal process [6].

When operated successfully, the EBPR process is a relatively inexpensive and environmentally sustainable option for P removal; however, the stability and reliability of EBPR can be a problem. It is widely known that EBPR plants may experience process upsets, deterioration in performance, and even failures, causing violations to discharge regulations. In some cases, external disturbances, such as high rainfall, excessive nitrate loading to the anaerobic reactor, or nutrient limitation, explains these process upsets. In other cases, microbial competition between PAOs and another group of organisms, known as the glycogen (non-polyphosphate) accumulating organisms (GAOs), has been hypothesized to be the cause of the degradation in P removal [7].

While the EBPR technology is well developed, the subsequent phosphorus release and recovery technologies need additional development [8].

Intensification of the P recovery processes and optimization of the fertilizer production conditions is necessary nowadays. Thus, it is important to develop an integrated approach for waste recycling.

The system of anaerobic digestion with heavy metals precipitation by biogenic hydrogen sulphide was developed for sewage sludge detoxification together with phosphogypsum (PG) [9].

Phosphogypsum is a byproduct from the processing of phosphate rock by the “wet acid method” of fertilizer production, which currently accounts for over 90% of phosphoric acid production. Because of trace impurities, this material has not been used to replace natural gypsum in the housing industry [10].

Currently, over 50 million tons of phosphogypsum are accumulated in Ukraine. Of all kinds of gypsum wastes of the Sumy region (Ukraine), the most appropriate is phosphogypsum. Currently, over 14 million tons of phosphogypsum are accumulated in the Sumy region. Phosphogypsum is produced in an amount of about 100 tons annually in PJSC “Sumykhimprom” [9].

Environmental pressures as well as increased land costs associated with stockpiling of phosphogypsum forces researchers to look for better utilization of this material.

Phosphogypsum is a multi-tonnage waste of phosphoric acid production. Solid waste is generated in the process of sulphuric acid decomposition of natural phosphate raw material and solid phase (calcium sulphate) separation from phosphoric acid solutions [9]. The reaction proceeds as follows:

Ca_5_(PO_4_)_3_F + 5Н_2_SO_4_ + nH_3_PO_4_ + mH_2_O → (n + 3)Н_3_PO_4_ + 5CaSO_4_·mH_2_O + НF (1)

The precipitate consists mainly of calcium sulphate dihydrate (CaSO_4_·2H_2_O) and contains impurities of phosphate, which is not decomposed, and silicates and it can be used in the anaerobic treatment system, which is one of the promising methods of phosphorus recovery.

This paper is focused on the biochemical treatment of sewage sludge with phosphogypsum under sulphate-reducing conditions with a P release process. To achieve the aim, the following tasks were set:

- Determination of the characteristics of phosphogypsum for use as a substrate of bacteria growth;
- Determining phosphogypsum’s influence on the process of phosphorus release from sewage sludge;
- Study the effect of the COD loading and phosphogypsum dosage on the metabolic activity of anaerobic microorganism groups in the process of phosphate release.

## 2. Materials and Methods

### 2.1. Sewage Sludge

Sewage sludge used in this work was collected from a wastewater treatment plant corresponding to a population of approximately 290,000 inhabitants located in Sumy city (Ukraine). Excess activated sludge collected from the thickener and flotation unit was used as feed for the anaerobic microbiological degradation pilot plant [9].

The main characteristics are listed in Table 1.

### 2.2. Phosphogypsum

As mentioned above, the phosphogypsum is used as the source of nutrients and macroelements for cultivated microorganisms used for the anaerobic digestion of sewage sludge with phosphorus release.

**Figure 1:**
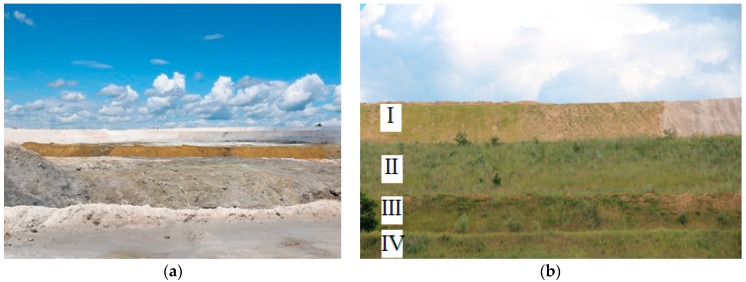
The phosphogypsum dump, Sumy region, Ukraine; (**a**) upper area of the dump massif with “fresh” phosphogypsum, (**b**) terraces (I, II, III, IV) of the phosphogypsum dump.

The phosphogypsum used in the experiments was sampled from the phosphogypsum dump (Figure 1), which is in operation in the Sumy region (Ukraine). The area of the dump is 492 m^2^ with a sanitary protection zone of 637 m^2^, the perimeter is about 1900 m, most of the phosphogypsum dump is preserved with loamy substrata and on the west side forms 4 terraces. To protect from drains, a protective reservoir, as a battery, was built near the dump on the north-west side.

Various parameters (mineral composition of the feedstock, serviceability of equipment, process discipline, fluent flow of production, etc.) can influence the quantitative content of impurities (Table 2).

The solubility of phosphogypsum is 2.1 g in 1 dm^3^ water (35 °C).

### 2.3. Methods of Investigation

The basic chemical composition of phosphogypsum was determined by X-ray fluorescence analysis (XRF). A SPECTRO iQ II (Ametek, Kleve, Germany) operating with a silicon drift detector (SDD, resolution of 145 eV at 10,000 pulses) was used.

Fourier transform infrared spectrophotometry (FTIR) measurements of samples of phosphogypsum from different dump terraces were carried out on a Bruker Alpha Platinum-ATR spectrometer (BRUKER OPTICS, Ettingen, Germany). A total of 24 scans were performed on each sample in the range of 4000–400 cm^−1^.

Mineralogical analyses of the phosphogypsum samples were carried out by X-ray diffraction (XRD) using a Bruker D2 Phaser (Bruker AXS, GmbH, Karlsruhe, Germany) in Bragg–Brentano geometry (configuration Theta-2Theta), using 1.54060 Å CuKα radiation, Ni Kβ filters, and a scintillation detector at a voltage of 30 kV and 10 mA current. Scan conditions: recording time about 2.5 h, a step size of 0.04° (2Θ) and step time of 5 s. The XRD patterns were processed using the software Diffrac.EVA v. 2.1 (Bruker AXS, GmbH, Karlsruhe, Germany). The ICDD PDF database (ICDD PDF—2 Release 2009) was utilized for phase identification.

Elemental analysis of the samples (liquid and solid phases) after the experiments was carried out on the X-ray fluorescence analyser Elvax Light SDD (Elvatech, Kiev, Ukraine). Limits of detection of impurities are not less than 10 ppm.

pH was analyzed by pX-meter pX-150 (ionometer) (Gomel Plant of Measuring Instruments, Gomel, Belarus).

A Merck^®^ spectroquant test kit (Merck KGaA, Darmstadt, Germany) was used to determine the total and soluble COD of the reactor. Prior to COD determination, all samples were acidified with concentrated HCl to pH = 2 in order to remove any dissolved sulphide.

The anaerobic bacterial colonies and extracellular structures were studied with electron microscopy. Micrographs of microbial preparations were prepared and processed using a digital image output system (SEO Scan ICX 285 AK-F IEE-1394) (Sumy Electron Optics, Sumy, Ukraine) and the morphometrical program SEO Image Lab 2.0 (Sumy Electron Optics, Sumy, Ukraine). Additional data was produced by scanning electron microscopy using a REMMA102 (Selmi, Sumy, Ukraine). Gram staining was carried out according to the conventional technique.

### 2.4. Anaerobic Microbiological Degradation Pilot Plant

The concept of our research is based on the use of phosphogypsum as a source of nutrients and macroelements (sulphates, phosphates etc.) for cultivated useful groups of microorganisms for anaerobic digestion of sewage sludge with phosphorus release.

This dephosphatation process consist of several important points: 1. the preliminary denitrification of the sewage sludge improves anaerobiosis in the phosphorus release stage; 2. phosphorus release is intensified by feeding phosphogypsum into the anaerobic bioreactor, which promotes the beginning of the sulphate reduction process on the principles of autoselection; 3. sedimentation of the solid phase; 4. the removal of phosphate ions from the liquid phase is expediently carried out in a separate purification unit by reagent precipitation.

**Figure 2:**
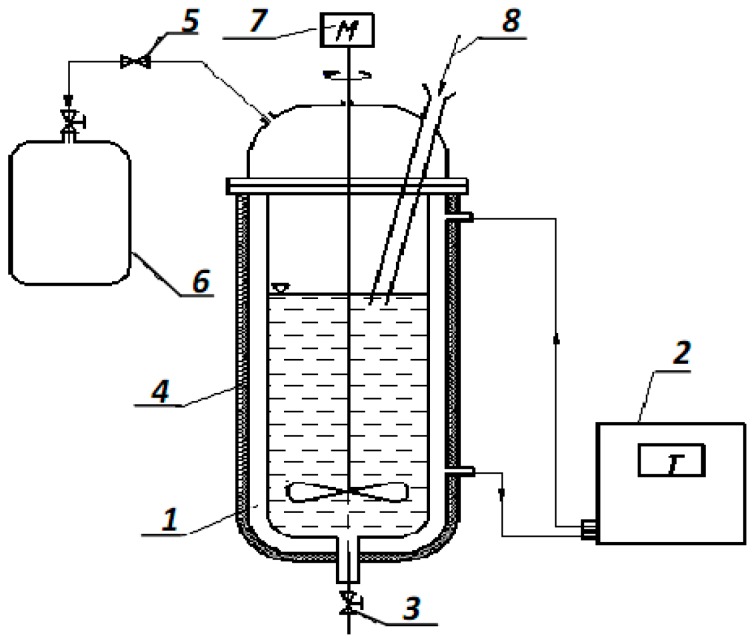
Experimental setup: 1—anaerobic bioreactor; 2—thermostat; 3—port for sludge removal; 4—insulation; 5—control valve; 6—biogas phase sampler; 7—mix device; 8—port for sewage sludge inflow and phosphogypsum loading.

The experimental setup consists of an anaerobic bioreactor where the release of phosphates takes place simultaneously with the reduction of sulphates (Figure 2).

The bioreactor (1) is a cylindrical anaerobic chamber for fermentation made from stainless steel, with a volume of 5 dm³. The working volume did not exceed 7/10 of the total volume. The chamber was covered with a heat-insulating film (4), under which heated water circulated through heat-resistant plastic pipes (313 K), which ensured the maintenance of a temperature of 309 K inside the bioreactor. The volume of circulating water was 3.6 dm^3^. To maintain the required temperature, the thermostat (2) Loip LT-108 (TZH-TS-01/8-100) (NV-Lab, Pavlodar, Kazakhstan) was used. In the lower part of the body of the bioreactor, there is an opening (3) for unloading anaerobically stabilized sludge. To ensure the tightness, the upper part of the bioreactor was tightly closed with a lid with an opening (8) for loading a new portion of the sludge, and also a branch pipe for evacuation of the gas phase with an adjustment valve (5). The substrate was fed to the bioreactor using a pump at a flow rate of 0.1 dm^3^/h. The hydraulic load was 0.013 m^3^/m^2^ h.

Sampling of gas samples was done in special sampling bags of inert plastic Teflon^®^ (6) with volumes of 1.0 and 6.0 dm^3^.

To mix the waste mixture in an anaerobic bioreactor, an agitator (7) with a motor power of 11 kV and a nominal speed of 0.42 r/s was installed.

Selection of fermented sewage sludge together with residual components of phosphogypsum for analysis was performed with a graduated glass in the amount of 0.05 dm^3^ (1.4% of the working volume of the bioreactor).

The digested sewage sludge outlet on the bottom of the reactor was used for sampling purposes. The initial pH of the system was from 6.5 to 7.5. The temperature (35 °C) in the bioreactor was kept constant with a temperature regulation device.

The monitored parameters were COD, pH level, concentration of phosphogypsum in the anaerobic system, concentrations of phosphorus compounds in the liquid and solid phases.

## 3. Results and Discussion

### 3.1. Phosphogypsum Composition Investigation

X-ray fluorescence analysis was used to describe the composition of phosphogypsum used in experiments with sewage sludge. The measurements included samples from all four terraces collected from the phosphogypsum dump located in the Sumy region (Ukraine). The results are summarized in the Table 3.

The achieved results approximately correlate with the numbers from the status report (Table 2). Differences in composition could be caused by the fact that the data listed in the status report represents the composition of fresh phosphogypsum material.

**Figure 3:**
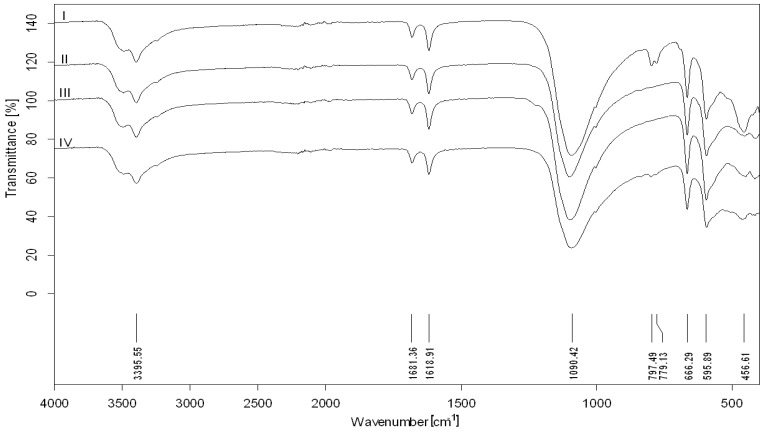
Infrared spectra of phosphogypsum (terraces I–IV).

The functional groups of phosphogypsum samples were characterized using FT-IR spectroscopy and their spectra are shown in Figure 3. The infrared spectra of studied phosphogypsum from terraces I–IV were almost identical.

The strong broad band at around wavenumbers 3395, 1681, and 1618 cm^−1^ was assigned to the hydroxyl functional groups. The two separate peaks at wavenumbers about 1681, 1619 cm^−1^, and intensely doubled peak at wavenumber 3395 cm^−1^ on the studied samples show that the –OH functional groups could be present due to bonded crystalline water in the hydrated form of phosphogypsum [12]. Bands at wavenumbers 1681 and 1619 cm^−1^ in the phosphogypsum samples are due to the presence of two types of water molecules in gypsum.

Sulphate compounds often occur in various parts of the environment due to rich natural sources and wide industrial usage. The tetrahedral sulphate anion exhibits nine normal modes [13] with a symmetric stretch ν 1 (1000 cm^−1^), a doubly degenerate symmetric bending ν 2 (500–400 cm^−1^), a triply degenerate asymmetric stretching ν 3 (1250–1050 cm^−1^), and a triply degenerate bending ν 4 (700–500 cm^−1^). The strong broad band at wavenumber 1090 cm^−1^ and deformations at wavenumbers 666, 596, and 457 cm^−1^ could be attributed to sulphate functional groups.

The FT-IR spectra of phosphogypsum were similar whatever the composition of the samples might be. The bands characteristic for phosphate groups are at wavenumbers 1120–1020, 960, 600–550, and 460 cm^−1^ [14]. These typical absorption deformations of the hydrogen phosphate ions were superimposed on those of the sulphate ion. However, from the obtained spectra it could be deduced that hydrogen phosphate has an absorption deformation area reflected at wavenumber 1250–1050 cm^−1^ that is accompanied by stretching to a width of the otherwise typical sharp asymmetric stretching of sulphate ions.

The silicon is characterised by the presence of SiO_4_ tetrahedral units. The typical IR spectrum of silica exhibits the presence of the two transverse optical modes of the Si–O–Si groups: the bending Si–O vibration identified around 800 cm^−1^ [15] and the asymmetric stretching mode of Si–O located in the range 1300–1000 cm^−1^ [16]. In this case, the asymmetric stretching mode of Si–O is covered by sulphate and phosphate functional groups with higher concentrations. Based on XRF study, it can be supposed that silicate is also present in phosphogypsum samples as impurities. The double peak adsorption deformations at wavenumbers 797 and 779 cm^−1^ visible only in sample from terrace I confirmed the presence of silica as a substance often found in the top of the earth’s surface.

**Figure 4:**
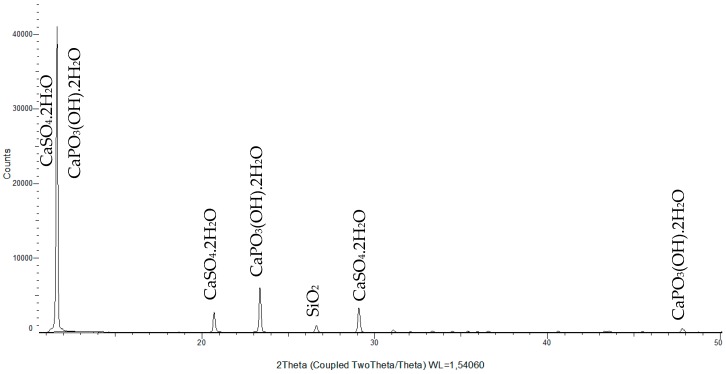
The X-ray diffraction pattern of phosphogypsum (terrace I).

The powder X-ray diffraction pattern of phosphogypsum (sample from terrace I) is shown in Figure 4. All diffraction patterns (terraces I–IV) were almost identical, except for quartz (SiO_2_) presence in the sample from terrace I, which confirmed the results from X-ray fluorescence analysis and FTIR spectra. The analysis of the pattern given by the diffraction showed that the sample contains calcium sulphate dihydrate (CaSO_4_·2H_2_O) in majority together with brushite (CaPO_3_(OH)·2H_2_O) and silicon oxide (SiO_2_). The samples are completely hydrated; the hemihydrate phase of calcium sulphate is missing in all measured samples.

### 3.2. The Phosphorus Release Process of Sewage Sludge and Phosphogypsum under Sulphate-Reducing Conditions

Previous research carried out in the laboratories of Sumy State University [9,17] proved the expediency of using phosphogypsum as a mineral substrate for the association of anaerobic microorganisms under sulphate-reducing conditions with biogas production. Using phosphogypsum as a mineral substrate for sulphate-reducing bacteria (SRB) growth has the following advantages:

- Low-cost raw material base that is rich in biogenic elements (calcium, sulphates, phosphorus, etc.);
- Sulphur compounds contained in the waste can be freely used by SRB as a mineral substrate for their growth, which is due to the high sulphate/sulphite ion affinity of microbial cells;
- Technogenic pressure reduction from chemical waste dumps on the environment.

The presence of a source of carbon and energy affects the biochemical process of separating phosphates from sewage sludge. Under anaerobic conditions, phosphor-mobilizing microorganisms (*Bacillus* (*B. megaterium*, *B. subtilis*), *Penicillium*, *Aspergillus*, etc.) convert organic compounds and transform slow-acting phosphorus compounds into a soluble form. The process of anaerobic phosphate release is enhanced by introducing phosphogypsum and, accordingly, stimulating the development of sulphate-reducing bacteria that secrete products of their own metabolism in the medium and indirectly stimulate the activity of phosphate-mobilization microorganisms, intensifying the allocation of phosphate ions in liquid phase.

**Figure 5:**
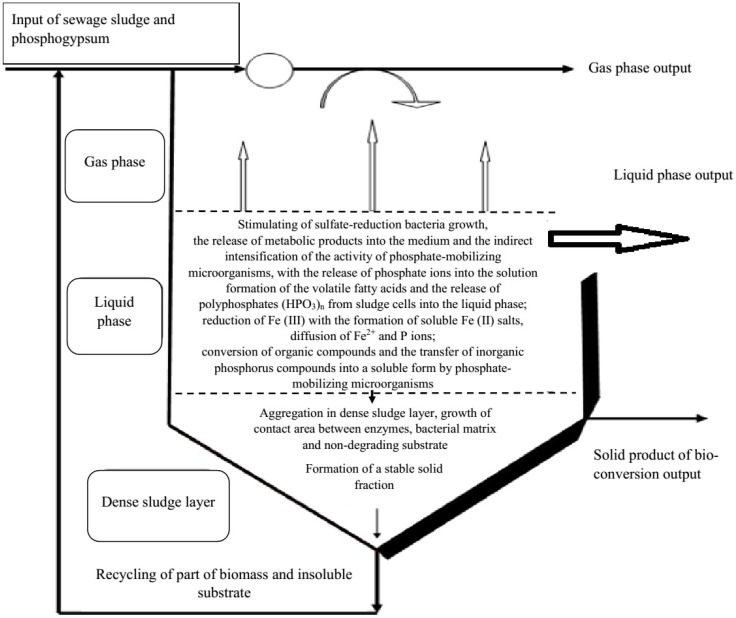
Formal model of the spatial distribution in the bioreactor in the phosphorus release process of sewage sludge and phosphogypsum under sulphate-reducing conditions.

Figure 5 shows a schematic model of the phosphorus release process under anaerobic stabilization of sewage sludge and phosphogypsum.

This model takes into account the process of returning part of the fermented substrate to the technological system and the flow of flocculation processes in the active sludge biomass.

**Figure 6:**
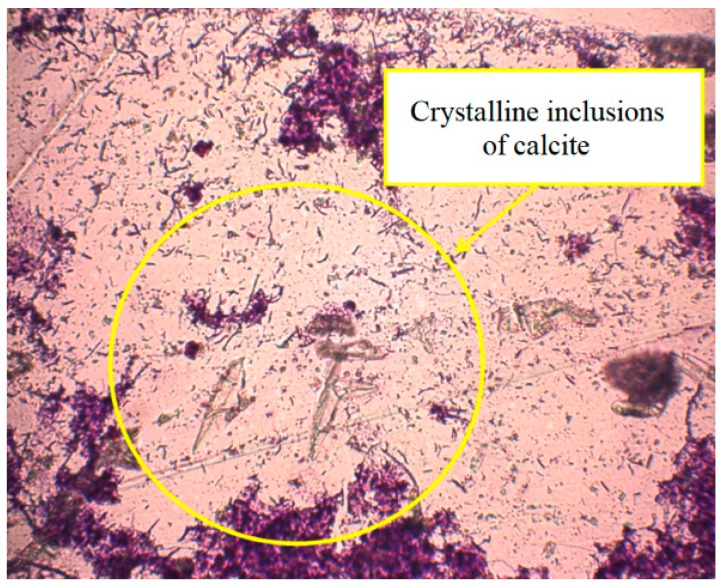
Crystalline structure formation during the process of digestion in the sewage sludge and phosphogypsum under sulphate-reducing conditions. Light microscopy. Gram staining. Magnification ×100.

Monitoring of the condition of the anaerobic sewage sludge showed that in the biochemical system there was the inclusion of semi-transparent small particles of the crystalline structure in the sludge flakes (Figure 6), which indicates the formation of a solid mineral phase. Its analysis showed the presence of calcium carbonate (calcite).

The association of microorganisms was dominated by acetatotrophs, which is due to incomplete oxidation of organic matter in acetates. Thus, the paramount importance for the stable operation of the system is the removal of the additionally formed volatile fatty acids. Otherwise, the pH can be reduced to 4.0.

**Figure 7:**
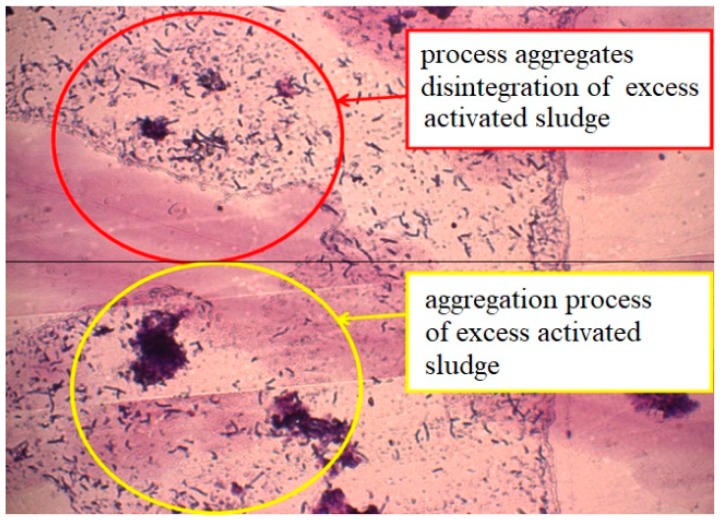
Formation and disintegration of sludge aggregates. Light microscopy. Gram staining. Magnification ×40.

Figure 7 is a spatial representation of the process of formation and decomposition of aggregates of sewage sludge. A significantly greater amount of fine mineral particles in the sludge flakes from the test sample compared to the control one affected the sedimentation properties of the silt, i.e., the properties of bio-flocculation.

In the process of anaerobic stabilization of sewage sludge and phosphogypsum, an association of microorganisms was formed which contained a considerable amount of sulphate-reducing bacteria. The population was determined as 1.0 × 10^10^ CFU/cm^3^ by electron microscopy. The obtained micrographs of fermented sewage sludge allowed observation of different forms of cellular aggregates with an increase in microorganism growth.

**Figure 8:**
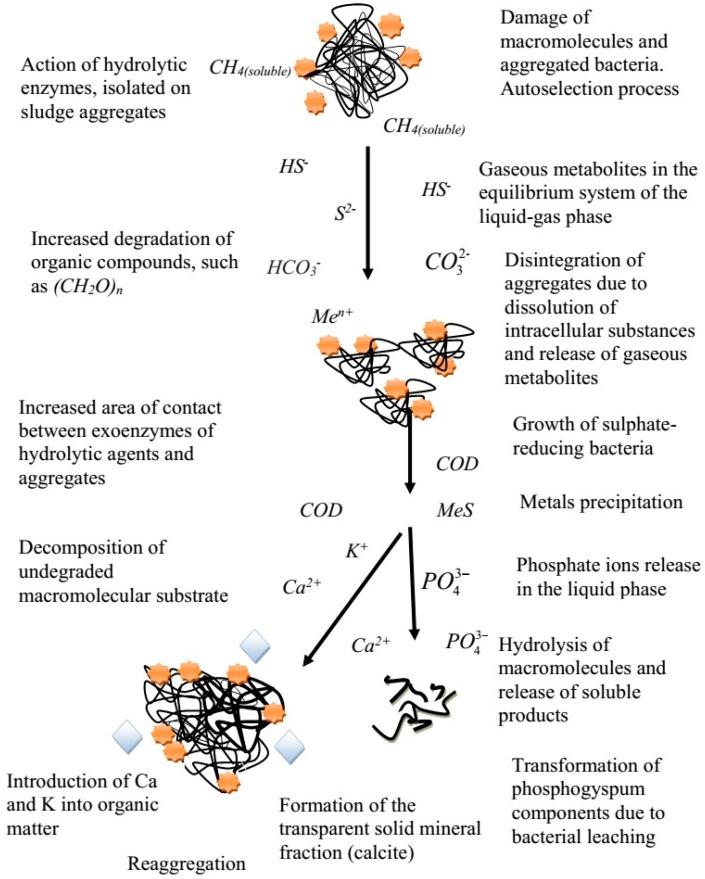
The general scheme of factors involved in the process of anaerobic conversion of sewage sludge and phosphogypsum under sulphate-reducing conditions.

The process of anaerobic phosphate release was enhanced by introducing phosphogypsum into the system and, accordingly, by stimulating the development of sulphate-reducing bacteria that secrete products of their own metabolism in the medium and indirectly stimulate the activity of phosphate-mobilizing microorganisms, intensifying the allocation of phosphate ions in solution (Figure 8). The involved factors have been reviewed in previous works [9,17]. Thus, the basic factors involved in the process of anaerobic conversion of sewage sludge and phosphogypsum under sulphate-reducing conditions are shown.

Substitution of metal complexes in microbial cells for Ca^2+^ phosphogypsum occurs in the course of ion exchange, and stimulates, in turn, the activity of exopolyphosphatase. As a result of this process, the liberated orthophosphate forms complexes with Ca^2+^ which are “ejected” from the cell into the external medium by means of transport systems.

Via the metabolism of sulphate-reducing bacteria, hydrogen sulphide is released, which affects the activity of enzyme systems of activated sludge cells. As a result, the separation of metal phosphate complexes occurs. The sulphide and hydrosulphide precipitates together with the metal ions in the sulphide solid fraction. For example, phosphates of iron are destroyed and iron sulphide is formed. In this case, the phosphate ions are released into the liquid phase. The next stage is precipitation of the phosphate ions by a reagent in the inorganic form that can be used as phosphorus fertilizer.

### 3.3. Effect of the COD Loading and the Phosphogypsum Dosage on the Process of Phosphate Release

**Figure 9:**
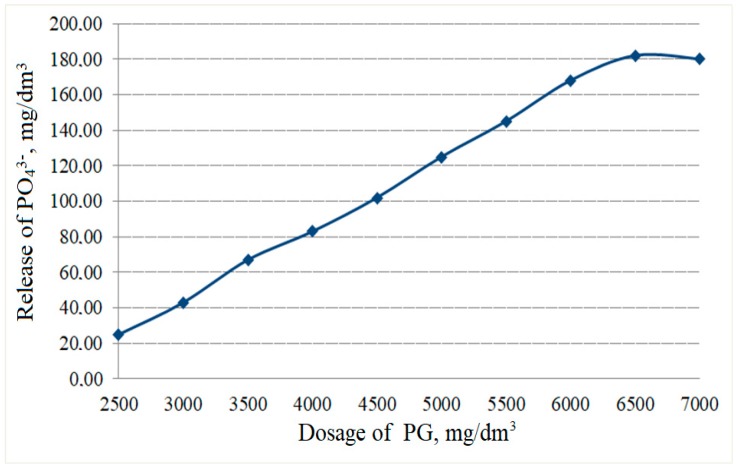
The relationship between phosphates release and the dosage of phosphogypsum (PG) under sulphate-reducing conditions.

The dose of phosphogypsum introduced into the anaerobic bioreactor, as a source of sulphate and other macro- and microelements (Ca, P, K, etc.) for the stimulation of sulphate-reducing bacterial growth, affected the amount of phosphate removal. The increase of phosphogypsum dosage had a close correlation with the release of phosphate ions (Figure 9).

It should be noted that this process was conducted with the biochemical conversion of phosphogypsum under anaerobic conditions. The obtained dependence of the process of phosphates release (M(Y)) on the concentration of phosphogypsum (X_1_) was approximated by the regression equation:

M(Y) = 0.0283X_1_^2^ + 20.014X_1_ + 2137.9 (R^2^ = 0.9837) (2)

According to our research (Figure 9), increasing phosphogypsum dosage up to 7000 mg/dm^3^ had no impact on phosphate release. Thus, a rational dose of phosphogypsum was 6500 mg/dm^3^.

Phosphate concentration in anaerobic bioreactors varies in a relatively wide range from 32 to 67 mg/dm^3^ PO_4_^3−^. The concentration of phosphate ions in the outflow was observed in the range from 112 to 179 mg/dm^3^ PO_4_^3−^. This means that as a result of the microorganism’s activity, a 2.5–5.0-fold increase in soluble phosphates concentration was observed with the stimulating action of the phosphogypsum additive.

Accordingly, the degree of phosphate ion increase (DI_P_) in solution was calculated by the formula:

(3) $$DI_{p}=\frac{\left(c_{1}-c_{0}\right)}{c_{1}}100\%,$$

where *c*_1_ and *c*_0_ are the concentrations of phosphates after and before treatment in the liquid phase, respectively.

**Figure 10:**
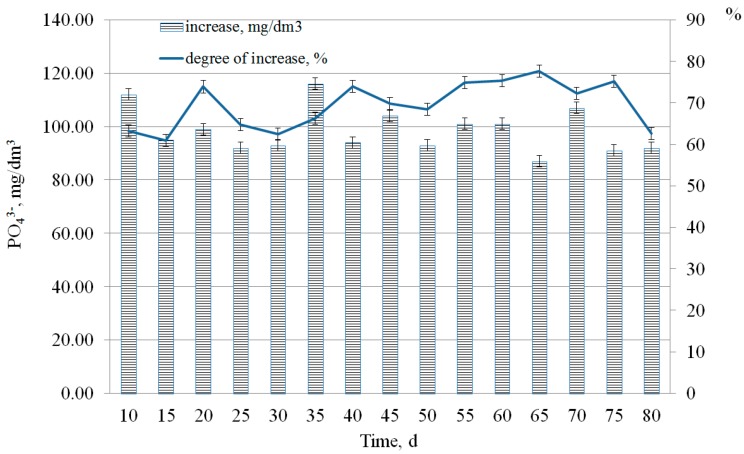
Increase in the concentration of phosphate in the liquid phase after treatment under sulphate-reducing conditions with phosphogypsum.

In Figure 10, it is shown that the degree of phosphate ions increase changed from 62% to 80%.

Together with the release of phosphates, the removal of the organic substrate was measured, which was expressed in changes of the COD. The initial COD of water extraction (1:1) of sewage sludge treated in an anaerobic reactor was in the range from 300 to 376 mg/dm^3^ O_2_. The final COD of a similar flow filtrate was much lower and was in the range from 96 to 147 mg/dm^3^ O_2_.

The maximum COD (181 mg/dm^3^ O_2_) was measured in the sludge at the initial stage of the dephosphatation. When the induction stage was passing, after 5 days in the stationary mode of operation (10–15 days), all measurements showed a value of COD below 145 mg/dm^3^ O_2_.

**Figure 11:**
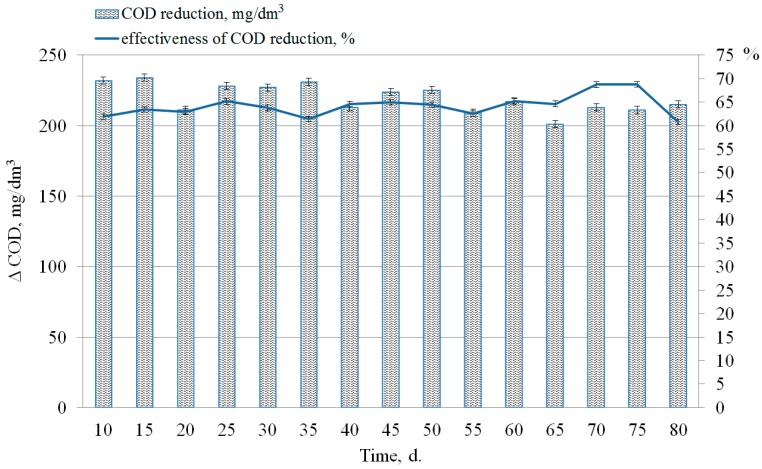
Determination of the level of COD reduction after anaerobic conversion of sewage sludge and phosphogypsum.

The effectiveness of COD reduction (Figure 11) can be calculated by the formula:

(4) $$E_{COD}=\frac{\left(COD_{0}-COD_{1}\right)}{COD_{0}}100\%,$$

where *COD*_0_ and *COD*_1_ are the values of COD before and after treatment in the liquid phase, respectively.

**Figure 12:**
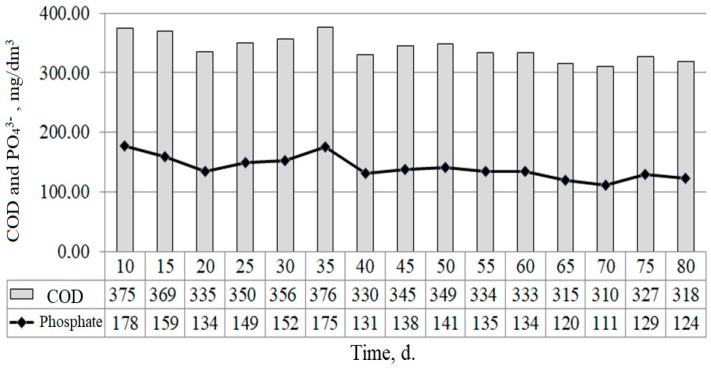
Dynamics of COD before anaerobic conversion and phosphate concentration after treatment.

The effect of organic compounds removal was expressed as a decrease in COD after the induction phase and it was not less than 60–70% (Figure 11). The value of COD in the output was 1.52 to 3.19 times lower than in the input. The variation of the measured values of the COD in the sewage sludge was reflected in changes in the concentration of phosphates (Figure 12).

Figure 12 shows that the COD level in sewage sludge undergoing treatment affects the level of phosphate anion release to solution.

**Figure 13:**
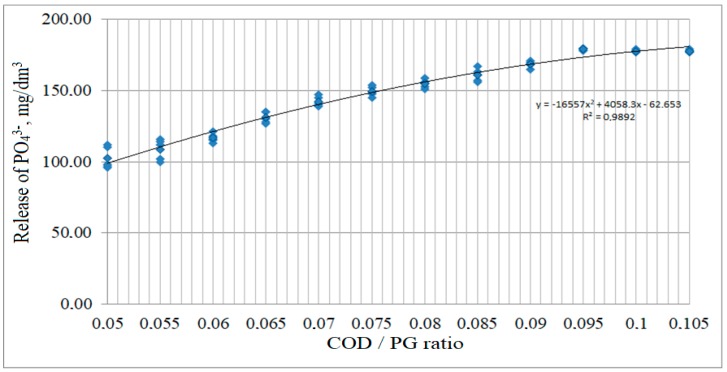
Influence of COD/PG ratio on phosphate release.

To understand the synergistic loading effect of the COD and the PG concentrations on the metabolic activity of the anaerobic microorganisms in the phosphate release process, the effect of the ratio of COD to the content of phosphogypsum dose on phosphate recovery was investigated (Figure 13). Thus, the increase in the ratio of COD/PG and the increase in the phosphate ions in solution corresponds to a decrease in phosphogypsum concentration from its initial dosage (6500 mg/dm^3^) via bacterial transformation.

Thus, according to the obtained results (Figure 13), the effect of the ratio of COD/PG (X_2_) on phosphate release (M(Y)) is approximated by the regression equation:

M(Y) = −16557X_2_^2^ + 4058.3X_2_ − 62.653 (R^2^ = 0.9892) (5)

Along with an increase in the ratio of COD/PG to 0.1, an increase in the phosphate ions in solution was observed. Further increasing the ratio of COD/PG had no effect on the concentration of phosphate ions.

## 4. Conclusions

A schematic model of the dephosphatation process under anaerobic stabilization of sewage sludge and phosphogypsum was developed, taking into account the process of returning part of the fermented substrate to the technological system and flocculation processes in the active sludge biomass. The model of anaerobic phosphate release shows the impact of phosphogypsum on the development of sulphate-reducing bacteria during the P release process in the bioreactor space with indirect intensification of phosphate-mobilizing microorganisms and thus the dissolution of phosphate ions.

The dose of phosphogypsum affected the amount of phosphate removal.

Increasing the phosphogypsum dosage had a close correlation with the release of phosphate ions. Phosphate concentration in anaerobic bioreactors varies in a relatively wide range from 32 to 67 mg/dm^3^ PO_4_^3−^. The concentration of phosphate ions in the outflow was observed in the range from 112 to 179 mg/dm^3^. Note that the initial phosphogypsum concentration is decreased by exposure to sulfate-reducing bacteria which use phosphogypsum as a substrate. This leads to a 2.5–5.0-fold increase in soluble phosphate release. Thus, a rational dose of phosphogypsum is 6500 mg/dm^3^.

The COD/PG ratio was investigated to understand its synergistic loading effect on phosphorus release during anaerobic treatment. Along with an increase the ratio of COD/PG to 0.1, an increase in soluble phosphate ions was observed. Further increasing the ratio of COD/PG did not affect the concentration of phosphate ions in solution. The next step will be developing the process of phosphate ion precipitation from the liquid phase for a complete P recovery process.

## Figures and Tables

**Table 1:** Main characteristics of the sewage sludge (mg/dm^3^) before treatment.

Total Solids	Volatile Solids	Total Suspended Solids	Volatile Suspended Solids	COD	PO_4_^3−^
2100–2130	1831–1867	2038–2040	1800–1960	300–376	32–67

**Table 2:** Composition of phosphogypsum in terms of oxides, % [11].

**CaO**	**SO_3_**	**A1_2_O_3_**	**Fe_2_O_3_**
30–42	44–52	0.3–5.0	0.2–2.0
**SiO_2_**	**F**	**P_2_O_5_**	**H_2_O**
0.3–1.0	0.1–1.0	1–4	25–40

**Table 3:** Composition of phosphogypsum in terms of oxides, %.

CaO	SO_3_	Al_2_O_3_	Fe_2_O_3_	SiO_2_	P_2_O_5_
28–36	52–61	0.1–0.2	0.1–0.3	1.2–19.9 *	0.3–1.3

* 19.9% of silicon oxide was determined in the sample from terrace I, which is probably caused by inflated particles of soil.
